# A simple fluorescent assay for cyromazine detection in raw milk by using CYR-stabilized G-quadruplex formation

**DOI:** 10.1039/c7ra12970j

**Published:** 2018-01-10

**Authors:** Haibo Xing, Wenchao Gu, Dang Xu, Fuxiang Tian, Linyun Yao, Zhenwei Wang, Xiaojun Hu

**Affiliations:** School of Chemical and Environmental Engineering, Shanghai Institute of Technology Shanghai 201418 China 267358918@qq.com; School of Perfume and Aroma Technology, Shanghai Institute of Technology Shanghai 201418 China; Putuo District Center for Disease Control and Prevention Shanghai China

## Abstract

A rapid biosensor for the detection of cyromazine in milk is reported based on a fluorescence quenching result. When an FAM labelled G-rich ssDNA Tcy2 is treated with cyromazine, it can form a G-quadruplex-CYR complex and cause a change in fluorescence. As a result, the presence of cyromazine can be determined by fluorescence quenching. This sensor is selective for the detection of cyromazine in raw milk and has a limit of detection of 0.68 ppb and a detection range from 0 to 200 ppb.

## Abstract 2

A rapid biosensor for the detection of cyromazine in milk is reported based on a fluorescence quenching result.

## Graphical abstract

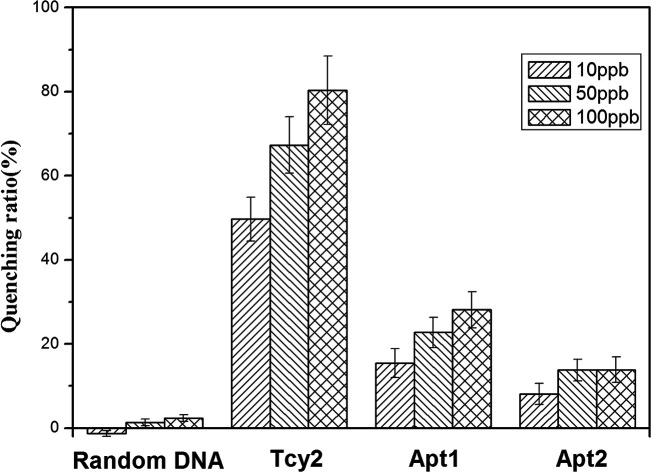

## 1. Introduction

Cyromazine (*N*-cyclopropyl-1,3,5-triazine-2,4,6-triamine, CYR) is a triazine pesticide, which is widely used in the livestock and poultry industry as an insect growth inhibitor for fly and maggot control.^1^ The overuse of cyromazine has been proved to cause problems of pollution by animal-derived food, and potential problems for environmental and human health, for example, mammary tumors in mice.^2,3^ Cyromazine can also be degraded to melamine, an industrial chemical compound which has been illegally added to food, animal feed, and even milk to increase the apparent protein content of products due to its high nitrogen content (66% by mass), and which causes kidney stones and kidney failure in high doses.^4,5^ Nowadays, the residues of cyromazine and melamine must be determined according to national standards before the derived food can be put on sale, so the field testing of cyromazine residues in animal-derived food, even raw milk, has become a hot issue. The U.S. FDA and China have set maximum residue limits (MRLs) for cyromazine in animal-derived food at 0.5 ppm, in contrast with the CAC limit of 1 ppm. Therefore, the development of an accurate, rapid and reliable method for the determination of cyromazine in raw milk is required to ensure food safety.

Nowadays, the main methods for the detection of cyromazine in raw milk and dairy products are as follows: gas chromatography-mass spectrometry (GC-MS) with the limits of quantification (LOQs) ranging from 10 to 100 ppb^6–8^ and liquid chromatography-mass spectrometry (LC-MS),^9–15^ ultra high performance liquid chromatography-high resolution mass spectrometry (UHPLC-MS/MS), and molecularly imprinted solid-phase extraction-ultra-performance liquid chromatography (MISPE-UPLC) with LOQs ranging from 0.05 ppm to 40 ppm.^16,17^ In the national standard of China (GB29704-2013), the residues of cyromazine and melamine are determinated by an ultra performance liquid chromatography-tandem mass spectrometric method (UPLC-MS). Although these methods are highly sensitive, most are either time consuming due to extensive pretreatment, or have a high cost due to the need for expensive instruments. Additionally, some new methods are also being developed for cyromazine and melamine monitoring, such as surface enhanced Raman spectroscopy (SERS), fluorescence, spectrophotometric absorption and chemiluminescence.^18–21^ Most of the rapid methods have been reported for melamine detection,^22–25^ and only a few are especially for cyromazine detection, such as the visual colorimetric detection of cyromazine in river water using gold nanoparticles.^21^ But there are no clear distinctions between cyromazine and its analogs. Therefore, simple, rapid, low-cost, easily operable methods are required for detecting cyromazine in milk and dairy products with good sensitivity and reliability.

Aptamers are artificial nucleic acid ligands that can bind their targets with high affinity and specificity, and they have therefore been widely used as recognition elements in the construction of biosensors.^26^ Hydrogen bonding between thymine and amino groups has been reported. With this hydrogen bonding, we have designed an aptamer-modified nanogold probe for melamine colorimetric detection using an ssDNA with 31 T bases, in which melamine can form a melamine–aptamer complex *via* hydrogen bonding. Thus, the resulting cationic polymer can aggregate the AuNPs and cause a remarkable change in color.^27^ A label-free AuNP based visual detection method for cyromazine in cucumbers using an ssDNA with ten T bases has also been reported.^28^ But these methods for the detection of cyromazine would be disturbed by analogs of cyromazine, such as melamine. Linear G-rich telomeric DNA strands can fold into G-quadruplex structures in the presence of monovalent cations such as K^+^ or Na^+^. G-quadruplex structures consist of two or more stacked planar G-tetrads.^29–31^ Inspired and encouraged by the similar structure and difference between cyromazine and melamine, we reported a sensitive detection system based on fluorescence quenching. When an FAM-labelled G-rich ssDNA Tcy2 was treated with cyromazine, it could form a G-quadruplex-CYR complex. Both the structure of the G-quadruplex formed by cyromazine and the occurrence of FRET between FAM and cyromazine can lead to a decrease in fluorescence, making the fluorescence quenching rate higher.

In this paper, an aptamer with 18 bases is designed to specifically combine with cyromazine, forming a G-quadruplex–CYR complex which results in a fluorescence quenching phenomenon, and a simple, sensitive and selective assay for cyromazine detection is proposed based on it.

## 2. Experimental

### 2.1 Materials and apparatus

We designed an ssDNA that is supposed to bind with cyromazine to form a G-quadruplex, and a random sequence was also used as a negative control. All the ssDNAs were labeled with 6-carboxyfluorescein (FAM) at the 5′ end and synthesized by Sangon Biotechnology Co, Ltd. (Shanghai, China), and the sequences are as follows:

Tcy2 5′-FAM-GGTTGGTTGGTTGGTTTT-3′ (18 bp)

Apt1 5′-FAM-TTTTTTTTTTTTTTTTTTTTTTTTTTTTTTT-3′ (31 bp)

Apt2 5′-FAM-GGGTAGGGCGGGTTGGG-3′ (17 bp)

Random DNA 5′-FAM-ATCGACATGTAGCCGATGGC-3′ (20 bp)

An F-4500 fluorescence spectrophotometer (Hitachi, Japan) was used to record the fluorescence intensity, with a response time of 0.5 s, PMT voltage of 700 V, scan speed of 1200 nm min^−1^, excitation wavelength of 480 nm, and excitation and emission slits of 10 nm. A time scan was operated when studying the kinetics of fluorescence quenching, with a scan time of 1200 s, excitation wavelength of 480 nm and emission wavelength of 520 nm.

A J-815 CD spectrometer (Jasco, Japan) was employed to characterize the structural changes in the oligonucleotides. The optical chamber (1 cm path length, 1 mL volume) was deoxygenated with dry purified nitrogen (99.99%) before use and kept under nitrogen atmosphere during the experiments. The scans (100 nm min^−1^) from 200 to 320 nm were taken three times at 1 nm intervals, then accumulated and averaged. The background of the buffer solution was subtracted from the CD data.

A thermostatic incubating device (Eppendorf, China) was used to carry out the quenching experiments at various temperatures.

The raw milk was purchased from a local nearby cattle farm. All reagents were of analytical grade. Milli-Q water (18 MV cm) was used in all experiments.

### 2.2 Pretreatment of samples

5 mL of 1% acetic acid was added into a 1.0 mL raw milk sample in a 10 mL centrifuge tube, then incubated for 5 min at room temperature after mixing well. The sample was centrifuged at 10 000 rpm for 10 min to separate the liquid component from the white opaque precipitation. In the following steps the final solution volumes of the samples were adjusted with ultrapure water.

### 2.3 Selection of cyromazine-binding ssDNA

Different ssDNAs with the same final concentration (25 nM) were individually dissolved in Tris–acetate buffer (10 mM, pH 8.0), and then various concentrations of cyromazine were added. A blank sample for each ssDNA was carried out in the absence of cyromazine. This step was done in order to calculate the *F*_0_. After incubation for 2 min at room temperature, the fluorescence intensity of each sample was measured and the quenching ratio, (*F*_0_ − *F*)/*F*_0_, was calculated, where *F*_0_ stands for the fluorescence intensity of FAM in the absence of cyromazine and *F* for the fluorescence intensity of FAM after the addition of cyromazine. The ssDNA which responded with the largest quenching ratio was chosen for subsequent study.

### 2.4 The kinetic curves of Tcy2 with cyromazine or K^+^

First the fluorescence intensity of each sample containing 25 nM Tcy2 was measured and then various concentrations of cyromazine or K^+^ were added individually. The fluorescence intensity of each sample was re-measured at 0, 10, 20, 30, 60, 120, 180, 240, 300, 360 seconds.

### 2.5 Investigation of the quenching mechanism

Quenching experiments at various temperatures (25 °C, 35 °C, 45 °C and 55 °C) and different cyromazine concentrations (0 ppb, 6 ppb, 15 ppb, 40 ppb, 100 ppb and 200 ppb) were carried out in Tris–acetate buffer (10 mM, pH 8.0) for 2 min and the fluorescence intensities in the absence and presence of cyromazine were recorded as *F*_0_ and *F*, respectively. A Stern–Volmer plot was generated by plotting *F*_0_/*F* against cyromazine concentration.

### 2.6 Sensitivity and selectivity of the detection of cyromazine

2.5 μL of 5 μM Tcy2 was firstly added into an appropriate volume of Tris–acetate buffer (10 mM, pH 8.0), and then various concentrations (from 0.5 to 200 ppb) of cyromazine were introduced into the above solution. The total volume of the final solution was fixed at 500 μL. After incubation for 2 min at room temperature, the fluorescence intensity was measured.

To determine the selectivity of the fluorescence assay, different veterinary drugs, including levamisole, abamectin, clopidol, amitraz, chloromycetin, thiamphenicol and terramycin, and different analogs of cyromazine and amino acids, such as ammonium hydroxide (NH_3_·H_2_O), urea, melamine, ammeline, ammelide, cyanuric acid, IgG, l-tyrosine, l-lysine, l-phenylalanine and l-valine, at a concentration of 50 ppb, were individually added to the sensor solution and the change in the fluorescence intensity was monitored.

## 3. Results and discussion

### 3.1 Principle of cyromazine biosensing using CYR-stabilized G-quadruplex formation

**Scheme 1:**
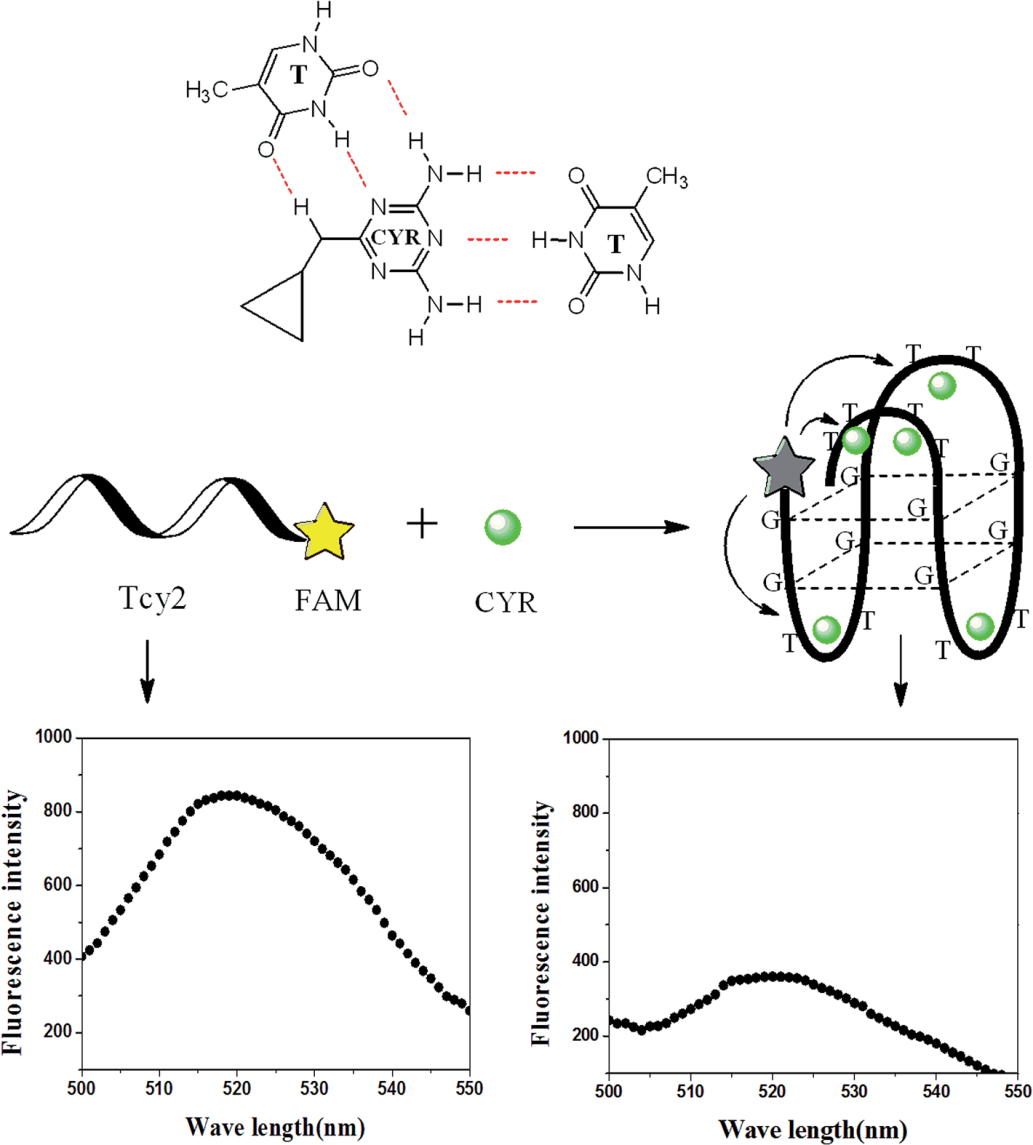
Schematic representation of the fluorescence assay for cyromazine detection based on the G-quadruplex combined with cyromazine.

The single-strand aptamer (ssDNA) of cyromazine, labelled with 6-carboxyfluorescein (FAM) at the 5′ end, is referred to as Tcy2. In the absence of cyromazine, ssDNA Tcy2 stays in its random coil conformation and the FAM shows strong fluorescence intensity. Whereas in the presence of cyromazine, a significant decrease in fluorescence intensity occurred. This fluorescence quenching phenomenon could be illustrated as follows (Scheme 1). In the absence of cyromazine, fluorescence of the labelled FAM could be excited by the exciting light, thus exhibiting strong fluorescence intensity. When the ssDNA was treated with other veterinary drugs that could not bind with it, it could not fold into the G-quadruplex. Under these conditions, the probability of intermolecular collisions between the fluorophore FAM and the drug would be considerably increased if the concentration of the drug was high enough.^32^ But, as Forster showed, the rate of fluorescence resonance energy transfer (FRET) depends on the inverse sixth power of the distance between the two fluorophores (FAM and drug),^33^ so the occurrence of FRET could not be guaranteed just by random collisions^34^ without the formation of a G-quadruplex. When the ssDNA was treated with other ions that have the ability to induce G-rich oligonucleotides to form a G-quadruplex, such as K^+^, the ssDNA could fold into the G-quadruplex which could bind some molecules with conjugated aromatic and potentially positively charged moieties. Compared with cyromazine, these molecules were implanted into the stacked G-quartets, not combined with the ssDNA. According to Kotch's report,^35^ the K(i)-stabilized G-quadruplex has a K–O distance of 2.80 Å, an O–O distance of 4.58 Å and a vertical separation of the G-quartets of 3.31 Å. In the present study, FAM was fixed at the 5′ end, and the FAM–K distance is approximately the same as that of K–O. Thus, based on the predicted secondary structure of the G-quadruplex formed from Tcy2, the FAM–CYR distance is much less than that of K–O. On the basis of Forster's theory,^33^ the longer distance could result in a dramatically lower FRET rate than that of cyromazine, hence ensuring the specificity of the cyromazine led fluorescence quenching. However, if the added ion was cyromazine, more than one molecule of cyromazine is in range so that the acceptor could get a reasonable energy transfer signal from the donor,^36,37^ thus making it possible to bring about the occurrence of FRET which results in fluorescence quenching.

**Fig. 1:**
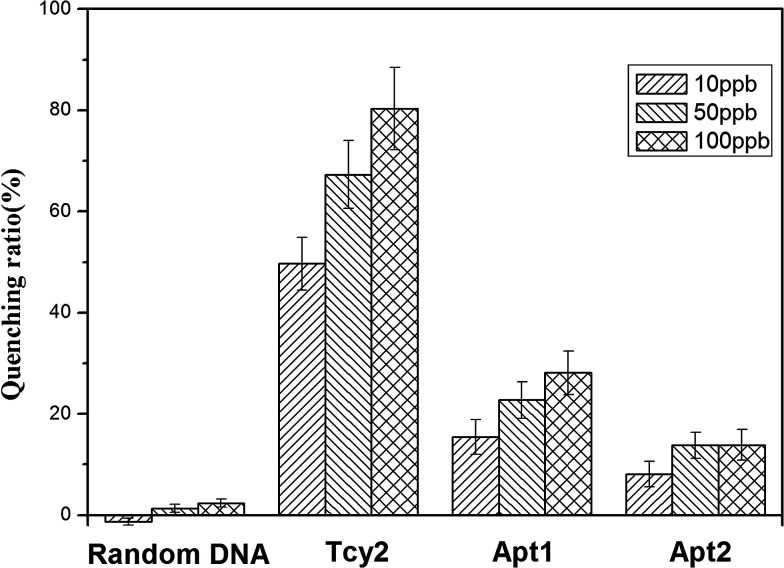
Quenching ratio caused by cyromazine at 10 ppb, 50 ppb and 100 ppb in individual systems containing different ssDNAs at the same concentration of 25 nM.

For the purpose of obtaining the most sensitive response and gaining a deeper insight into the mechanism, several ssDNAs (Apt1, Apt2) are supposed to bind with cyromazine to form a G-quadruplex according to previous reports^38^ and a randomly designed sequence (random DNA) was studied. As shown in Fig. 1, no obvious quenching effect was obtained when the FAM-labelled Random DNA was treated with cyromazine at low or high concentrations. This result indicates that the quenching phenomenon appearing in our study was actually aroused by some reaction between cyromazine and the CYR-binding ssDNA, thus disproving the hypothesis that the cyromazine may cause the fluorescence quenching directly.

### 3.2 Interactions between the ssDNA and cyromazine

**Fig. 2:**
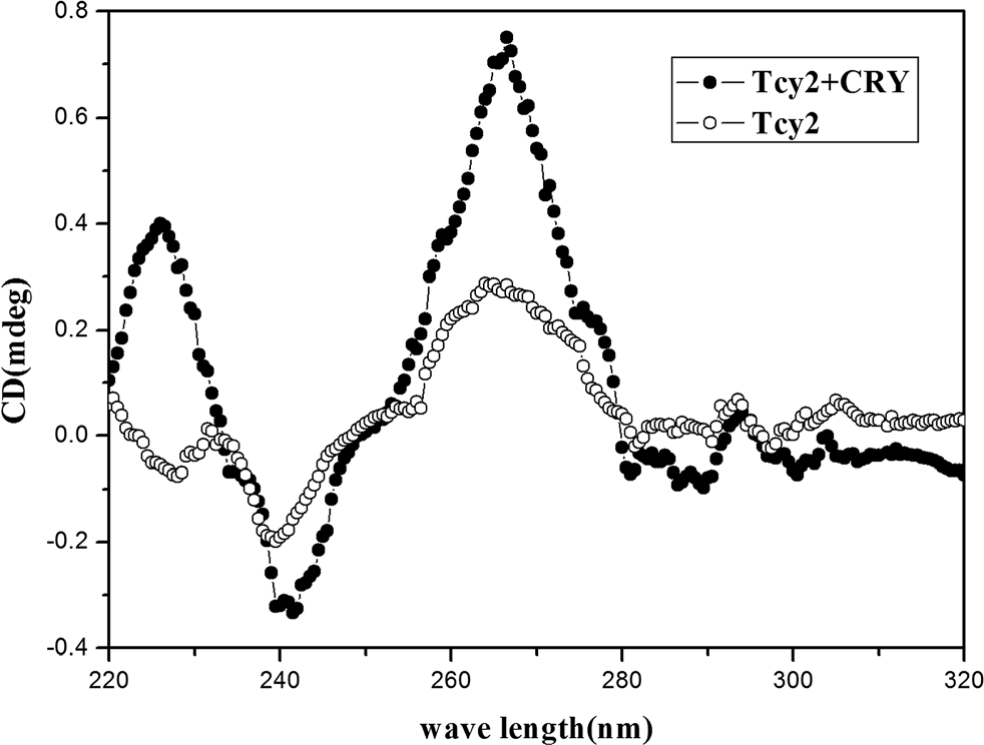
CD spectra of 1 μM Tcy2 in the absence and in the presence of 1 ppm of cyromazine. The cyromazine treatment reaction was performed in MOPS buffer (10 mM, pH 7.0) for 5 min at room temperature.

Circular dichroism (CD) is a commonly used method for the analysis of conformational change in nucleic acid aptamer reactions. Thus, CD provides a way to confirm that some specific structure was formed when cyromazine reacted with the ssDNA Tcy2. The results showed that an enhancement in the negative peak around 240 nm and the positive peak around 260 nm was observed upon the addition of 1 ppm of cyromazine (Fig. 2). As it has been reported that a typical CD spectrum of a “parallel” G-quadruplex structure has a negative peak at around 240 nm and a positive peak near 260 nm,^39–41^ the results indicated that a parallel G-quadruplex was formed with the presence of cyromazine.

### 3.3 The kinetic curves of Tcy2 with cyromazine or K^+^

**Fig. 3:**
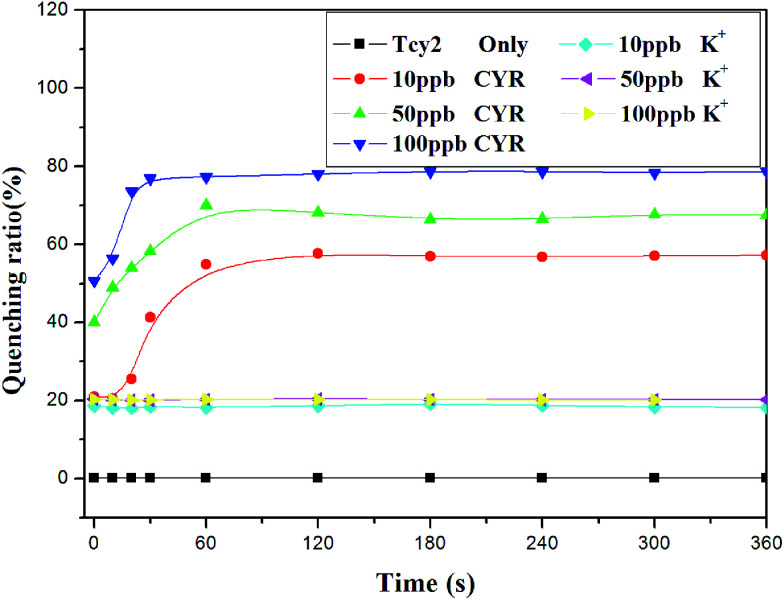
Kinetic curves of 25 nM Tcy2 in the absence and in the presence of cyromazine (10 ppb, 50 ppb, 100 ppb).

Time plays an important role in the cyromazine–oligonucleotide reaction, so the fluorescence quenching of this sensor system was tested at various reaction times. As shown in Fig. 3, all the FAM-labelled ssDNAs show fluorescence intensity at the same level before being treated with molecules. After various concentrations of cyromazine were added, different fluorescence intensity changes occurred, while K^+^ could not bring about notable changes for a long time. It could be observed that cyromazine caused obvious changes quickly even at low concentration, and the higher the concentration of cyromazine, the more obvious the fluorescence quenching. As all the fluorescence intensities tended to remain constant after 120 s, 2 min or a little longer was consequently chosen as the optimum reaction time. Although K^+^ might induce the ssDNA to form a G-quadruplex structure, the FRET between K^+^ and FAM was much lower than that between cyromazine and FAM, and it hardly changed as time went on.

### 3.4 Investigation of the quenching mechanism

**Fig. 4:**
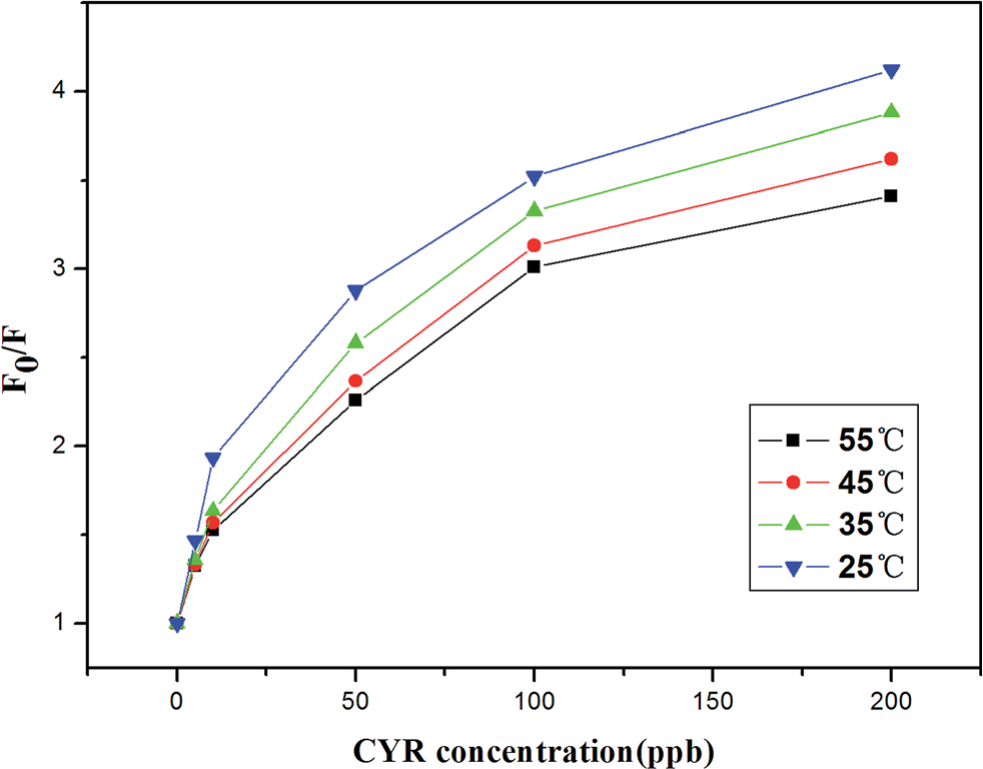
Stern–Volmer curves for the fluorescence quenching by cyromazine of different concentrations (0 ppb, 5 ppb, 10 ppb, 50 ppb, 100 ppb and 200 ppb) at four different temperatures (25 °C, 35 °C, 45 °C and 55 °C). The concentration of Tcy2 is 25 nM.

The well-known Stern–Volmer equation, *F*_0_/*F* = 1 + *K*_sv_[Q], was applied to investigate the quenching mechanism and to determine whether it is a dynamic or static process, where *F*_0_ and *F* are the fluorescence intensities in the absence and presence of cyromazine, respectively, *K*_sv_ is the Stern–Volmer quenching constant and [Q] is the quencher (*i.e.* cyromazine) concentration,^42^ and the temperature dependence of the quenching was examined.^43^ Quenching experiments at various temperatures and different cyromazine concentrations were carried out and a Stern–Volmer plot was generated (Fig. 4). The results show that the value of *F*_0_/*F* at each concentration of cyromazine decreased with an increase in temperature and the curvature went down at high cyromazine concentration. So from the results two conclusions could be inferred. First, when the temperature increased, the quenching constant *K*_sv_ decreased, so the quenching in our study is static quenching.^44^ Second, the downward curvature reveals the inaccessibility of the fluorophore fraction to the quencher at high cyromazine concentration. This verifies our conjecture that the fluorescence quenching is primarily caused by the formation of a CYR-stabilized G-quadruplex. When the cyromazine concentrations increased to a high level, the excess cyromazine could not induce any more Tcy2 to form a G-quadruplex because the amount of G-quadruplex was fixed by the concentration of Tcy2.

### 3.5 Sensitivity and selectivity of the detection of cyromazine

**Fig. 5:**
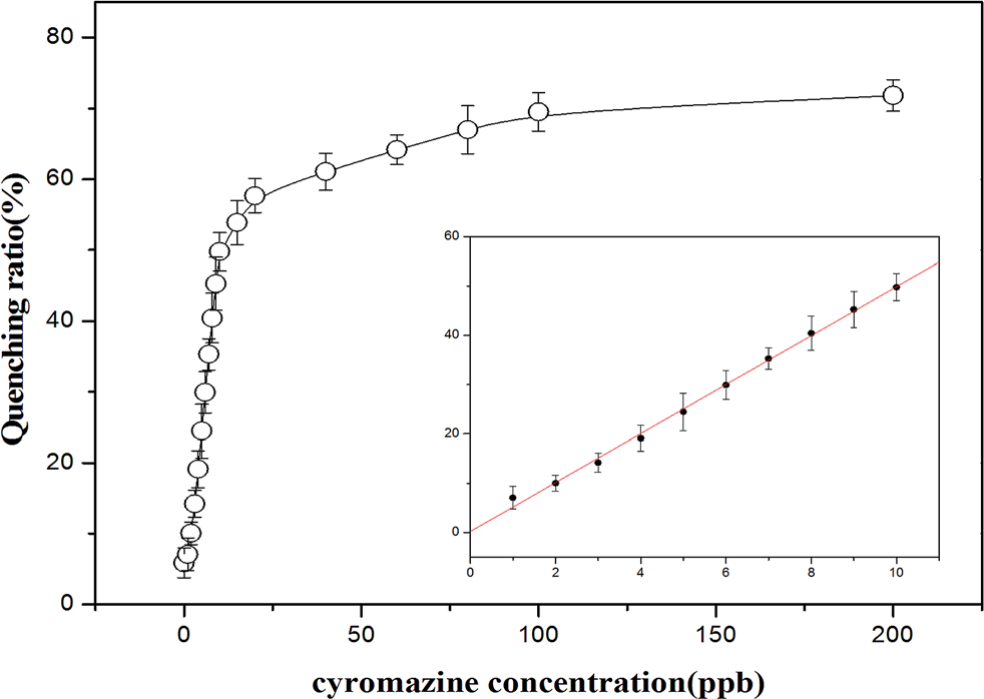
Calibration curve of the sensing system for cyromazine. The concentrations of cyromazine are 0, 1, 2, 3, 4, 5, 6, 7, 8, 9, 10, 15, 20, 40, 60, 80, 100 and 200 ppb, the concentration of Tcy2 is 25 nM.

The fluorescence signals for different concentrations of cyromazine were measured to explore the sensitivity of this assay. Fig. 5 depicts the quenching ratio plotted against the concentration of cyromazine by fitting it to a Hill plot with a correlation coefficient of 0.985. A linear correlation exists between the ratio values and the concentration of cyromazine over the range 0.1 to 1.0 mM, with a correlation coefficient of 0.999. The equation is *y* = 4.962*x* + 0.2517. This sensor has a limit of detection (LOD) of 0.68 ppb, which was calculated from 3 s per slope.^45,46^ As the maximum contamination level for cyromazine in raw milk, as defined by the U.S. FDA and China, is 50 ppb or 300 nM, the assay has high sensitivity for the quantitative analysis of cyromazine in raw milk.

**Fig. 6:**
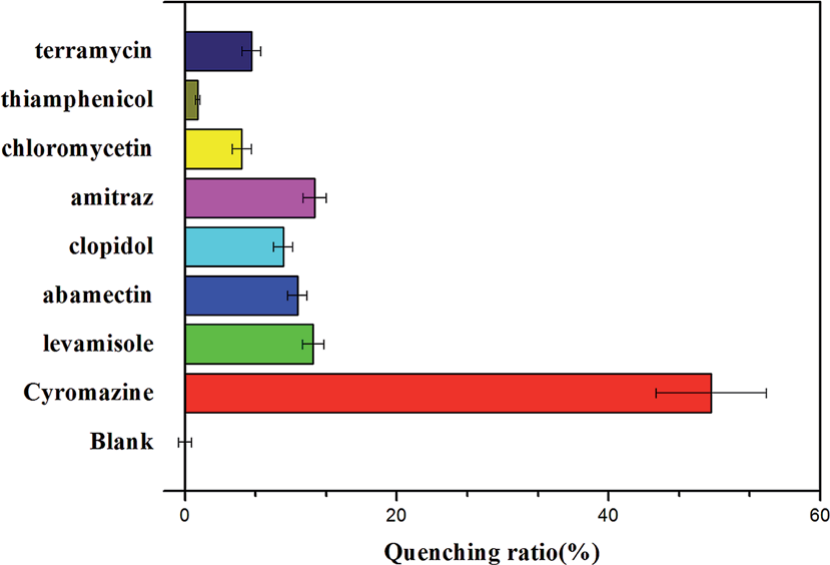
Selectivity of the assay for cyromazine detection over other veterinary drug concentrations: cyromazine (15 ppb); levamisole, abamectin, clopidol, amitraz, chloromycetin, thiamphenicol, terramycin (50 ppb). The concentration of Tcy2 is 25 nM.

**Fig. 7:**
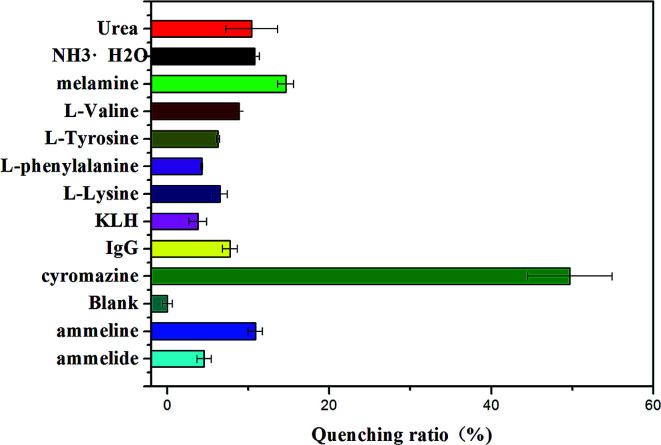
Selectivity of the assay for cyromazine detection over other compound concentrations: cyromazine (15 ppb); others (50 ppb). The concentration of Tcy2 is 25 nM.

The selectivity of this assay for the determination of cyromazine was also investigated. Control experiments were performed using other common veterinary drugs, such as levamisole, abamectin, clopidol, amitraz, chloromycetin, thiamphenicol and terramycin. Eleven competitive compounds with a similar structure or which occur in raw milk, ammonium hydroxide (NH_3_·H_2_O), urea, melamine, ammeline, ammelide, cyanuric acid, IgG, l-tyrosine, l-lysine, l-phenylalanine and l-valine, were also individually added to the sensor. As shown in Fig. 6, we can see that these nonspecific veterinary drugs did not lead to characteristic changes in fluorescence even at high concentrations. Fig. 7 shows that there were no evident changes in fluorescence for the mixture of the sensor solution with the eleven compounds, except with cyromazine. Although there is also hydrogen bonding between thymine and the amino group of some of the compounds above, they would not form the same G-quadruplex and cause a remarkable change in fluorescence. These results demonstrate that all the other compounds display slight or negligible interference with the detection of cyromazine by this biosensor.

### 3.6 Detection of cyromazine in raw milk samples

Before sample pretreatment, we added different amounts of cyromazine to the raw milk. This proposed method was repeated 3 times for each sample and HPLC-MS was also applied to analyse the cyromazine in the raw milk samples. As is shown in Table 1, the recoveries are between 90% and 120% by this method, while the recoveries are between 98% and 102% by HPLC-MS. This indicates that this method of using a CYR-stabilized G-quadruplex as a probe for the rapid detection of cyromazine in raw milk is feasible.

Application of this method and HPLC-MS to detect the cyromazine in raw milk samples spiked with different amounts of cyromazine

**Table:** 

Sample	Concentration of cyromazine (ppm)		Recovery (100%)
	Added	Found (G-quadruplex)	
1	0.100	0.12 (±0.02)	120
2	0.200	0.19 (±0.02)	95
3	0.500	0.53 (±0.06)	106
4	1.000	1.06 (±0.08)	106

**Table:** 

Sample	Concentration of cyromazine (ppm)		Recovery (100%)
	Added	Found (HPLC-MS)	
1	0.100	0.10	100
2	0.200	0.20	100
3	0.500	0.49	98
4	1.000	1.02	102

## 4. Conclusions

In summary, we have developed a rapid, sensitive and selective fluorescence assay for the detection of cyromazine in raw milk by using an FAM-labelled G-rich oligonucleotide Tcy2 as a recognition probe. This assay is based on the formation of a CYR-stabilized G-quadruplex and the fluorescence resonance energy transfer (FRET) between cyromazine and FAM. In the absence of cyromazine, ssDNA Tcy2 stays in its random coil conformation, accompanied with strong fluorescence intensity, but once cyromazine was added into this system, Tcy2 formed into a G-quadruplex structure and made the FAM closed to cyromazine, enabling the FRET between them, leading to a remarkable fluorescence quenching phenomenon. The LOD of this method for the detection of cyromazine in raw milk is as low as 0.68 ppb. Moreover, compared with traditional cyromazine sensors, this assay is facile and convenient without involving expensive sophisticated instruments and long response times. So we hope that this type of detection method, which holds great practicality for real-time and on-site cyromazine detection in environmental monitoring, will be realized for the detection of other molecules in food samples.

## Conflicts of interest

The authors declare that they have no conflict of interest. All procedures performed in studies involving human participants were in accordance with the ethical standards of the institutional and/or national research committee and with the 1964 Helsinki declaration and its later amendments or comparable ethical standards. This article does not contain any studies with animals performed by any of the authors. Informed consent was obtained from all individual participants included in the study.

## Supplementary Material
